# COHORT DIFFERENCES IN PENSION AND JOB SECURITY OF OLDER UNION WORKERS: EVIDENCE FROM HRS

**DOI:** 10.1093/geroni/igae098.0463

**Published:** 2024-12-31

**Authors:** Yun taek Oh

**Affiliations:** University of Nevada Reno, Reno, Nevada, United States

## Abstract

The influence of the declining number of unions and their bargaining power on older workers’ quality of work lives, such as pension eligibility and job security, is an understudied field. I use the first six waves of the initial HRS cohort (born 1931-1936, recruited in 1992, N = 3,134) and Mid Baby Boomers (MBB, born 1954-1959, recruited in 2010, N = 2,886) from the Health and Retirement Study (HRS) from 1992 to 2020. I examine the changes in the association between union membership and the quality of later work lives by using the interaction terms between birth cohorts, union membership, and employment sector (private or public). The results from logistic regressions show that the proportion of pension eligibility from current jobs declined over time while the expectation of job loss at ages 57-62 is stable for union members. Furthermore, the predicted probability of actual job loss increased among non-union workers in the private sector while that for union workers was unchanged. These results suggest that the union’s declining bargaining power may influenced pension eligibility although expectations and actual job security remain unchanged for union workers. The results from competing risk analysis show that MBB union workers are more likely to change job titles while less likely to move to different employers or to directly exit the workforce than the previous cohort. This result suggests the prevalence of bridge employment among union workers as well as the preference changes in the type of bridge employment.

